# The enhanced Aussie Optimism Positive Thinking Skills Program: The relationship between internalizing symptoms and family functioning in children aged 9–11 years old

**DOI:** 10.3389/fpsyg.2015.00504

**Published:** 2015-04-30

**Authors:** Patricia Kennedy, Rosanna M. Rooney, Robert T. Kane, Sharinaz Hassan, Monique Nesa

**Affiliations:** School of Psychology and Speech Pathology, Faculty of Health Sciences, Curtin UniversityPerth, WA, Australia

**Keywords:** AO-PTS, internalizing symptoms, family functioning, protective factors, child perceptions

## Abstract

The family context plays a critical role in the health of the child. This was the first study to examine the usefulness of the General Functioning subscale of the Family Assessment Device (FAD-GF) in assessing family functioning and its relationship to internalizing symptoms in school-aged children aged between 9 and 11 years of age. Eight hundred and forty-seven year 4 and 5 students from 13 schools (607 intervention students, and 240 control students) participated in the Aussie Optimism Positive Thinking Skills Program (AO-PTS) – a universal school-based program targeting internalizing symptoms. Students rated how ‘healthy’ they perceived their family to be at pre-test and at 6-months follow-up. Although some aspects of validity and reliability could be improved, results indicated that perceptions of family functioning at pre-test were predictive of internalizing symptoms at the 6-months follow-up. The FAD-GF therefore showed promise as a potential measure of family functioning for children as young as 9 years old. Regardless of children’s pre-test levels of perceived family functioning, no intervention effects were found on the anxiety and depression scales; this finding suggests that child perceptions of family functioning may act as a general protective factor against internalizing symptomology.

## Introduction

Internalizing symptoms and disorders refer to a range of difficulties primarily characterized by a disturbance in mood or emotion that include feelings of anxiety and depression (Hughes and Gullone, 2008). In children, internalizing symptoms and disorders have become an increasing mental health concern and are thought to affect one in every seven school aged child (Bayer et al., 2011) with a median age of onset of 11 years (Kessler et al., 2005). Internalizing disorders are estimated to affect 10–15% of preschool children aged between 18 months and 5 years (Bayer et al., 2011). Moreover, 6–8% of children aged between 6 and 12 years suffer from depression, while 10–20% of these children suffer from anxiety (Briggs-Gowan et al., 2004; Rapee et al., 2009). An additional 40–60% of these children will also meet the criteria for at least one other anxiety disorder during their childhood (Rapee et al., 2009). Internalizing symptoms and disorders have been shown to persist through childhood and into adolescents causing considerable adverse effects in terms of psycho-emotional functioning (e.g., low self-esteem) psychosocial functioning (e.g., poor school competence, problems with peer relationships; Bayer and Sanson, 2003; Bayer et al., 2011; Teubert and Pinquart, 2011) and family functioning (Rapee et al., 2009; Drake and Ginsburg, 2012).

The family context provides an important setting for the child’s social and emotional wellbeing, and research has demonstrated that family functioning is a key contributor to a child’s wellbeing (Bögels and Brechman-Toussaint, 2006; McClelan and Cohen, 2006; Hughes et al., 2008; Rapee et al., 2009; Rapee, 2012). In the current literature, the term “family functioning” is often used as an umbrella term to describe a range of family and parent constructs that reflect the family structure and the nature of the relationships within the family including parental psychopathology (Hughes et al., 2008), family cohesion and adaptability (Drake and Ginsburg, 2012), family conflict (including marital conflict; Bögels and Brechman-Toussaint, 2006), marital quality (Schermerhorn et al., 2012), parenting styles (e.g., over protection, rejection; McLeod et al., 2007), parental behaviors (e.g., low warmth, over-control, criticism, modeling; McLeod et al., 2007), parental stress, parental bonding, single parenthood and the accompanying socioeconomic problems (Moffitt et al., 2007), stressful life events, perceptions of low social supports, and emotional warmth (Wilhelm et al., 2000).

### Prevention

Research indicates that greater reductions in anxiety are reported by children who undergo active interventions compared to children who do not (Rapee et al., 2009). There has been a recent surge in prevention research for children, with a particular interest in identifying the strategies that make effective interventions accessible to children ‘at risk.’ In their review of the efficacy of Australian school-based prevention and early intervention programs for anxiety and depression, Neil and Christensen (2007) indicate that improvements in symptoms of anxiety and depression occurred in 60% of universal programs targeting anxiety and 58% of universal programs targeting depression. In a more recent meta-analysis, Durlak et al. (2011) found that school-based universal intervention programs significantly reduced conduct and internalizing problems in children. These effects remained significant for a minimum of 6 months post-intervention. Short-, mid-, and long-term (up to 30 and 36 months) effectiveness of school-based universal approaches have been reported for the Aussie Optimism Positive Thinking Skills Program (Rooney et al., 2004, 2013; Morrison et al., 2013) and *FRIENDS* (Shochet et al., 2001). Lock and Barrett (2003) further evaluated the longitudinal effects of universal school-based interventions on child anxiety. This study focused on two different cohorts of children: primary school children aged 9–10 years (*N* = 336) and secondary school children aged 14–16 years (*N* = 401). Compared to the 14–16 years olds, the 9–10 years olds reported the greatest reduction in anxiety symptoms and the largest increase in coping skills, indicating that 9–10 years of age might be the optimal time for prevention intervention. The Aussie Optimism Positive Thinking Skills Program is an Australian universal program implemented within schools that targets large numbers of children, regardless of risk status, with the aim of preventing the incidence of internalizing problems.

Overall, the literature indicates that various factors such as parental involvement in the program (e.g., attendance, satisfaction; Essau, 2005), type of intervention (e.g., CBT, IPT, coping strategies, resilience building, psycho-education), intervention duration, methodological impurities (e.g., lack of randomized control groups, small sample sizes), child factors (e.g., willingness, age, ethnicity, gender, accuracy of self-reports, perfectionism, coping styles), program content (e.g., problem solving, social skills, homework; Stice et al., 2009) and provider features (e.g., teachers, psychologists) may act as mediating factors and influence the efficacy of universal prevention programs targeting internalizing disorders in children (Stice et al., 2009). Given family functioning has been connected to a child’s wellbeing, assessing the impact of how healthy or unhealthy a child perceives their family on the efficacy of prevention programs such as the AO-PTS Program remains to be clarified. A review of the literature failed to clarify whether other variables, such as family functioning, influences the treatment outcomes and efficacy of universal prevention programs. Surprisingly, no study to date has investigated the influence of family functioning on the efficacy of internalizing prevention programs in children as young as 9–10 years of age.

Research has demonstrated that higher levels of family dysfunction have negative consequences for children (Bayer and Sanson, 2003; Hughes et al., 2008; Rapee et al., 2009). Researchers in general indicate that families of children with anxiety or depression tend to report greater family dysfunction than families of children with no disorders (Hughes et al., 2008). More specifically, problematic family functioning or dysfunction has been found to be a correlate of childhood anxiety (Crawford and Manassis, 2002) and the evidence suggests that children who report elevated levels of internalizing symptoms perceive their family more negatively and dysfunctional compared to children who report normal levels of internalizing symptoms (Stark et al., 1990; Crawford and Manassis, 2002; Nomura et al., 2002; Hughes et al., 2008).

One large population study of over 3000 young people aged between 14 and 24 years old, who were followed for 10 years identified family dysfunction as one of the risk-factors in the development of generalized anxiety disorder (Beesdo et al., 2010). This study indicates the need to evaluate the role of family functioning in assessing children’s internalizing behaviors. The interaction between child temperament and parental factors such as attachment are thought to play a significant role in the development and maintenance of internalizing symptoms (Crawford and Manassis, 2002; Dadds and Roth, 2008). Surprisingly, few studies have evaluated the overall health and general functioning of the family and its specific relationship with internalizing symptoms.

When assessing family functioning, researchers in general agree that using multiple informants limits method bias by increasing the reliability and validity of the assessment. This is considered the ‘gold standard’ (Taber, 2010). Despite this focus on multiple informants, large discrepancies continue to exist (Reys and Kazdin, 2005). More recently, the potential clinical utility of children’s perceptions appears to be gaining support among researchers (Jager et al., 2012). In a study of family conflict, children’s perception of parental fighting was a better predictor of children’s functioning than changes in family structure (Cummings, 1994). The consistent finding that parental psychopathology is strongly correlated with child internalizing suggests that it is not the psychopathology itself that increases risk to the child, rather it is how the child *perceives* the disturbance to the family that causes distress (Jager et al., 2012).

Researchers who study family functioning commonly use well established measures including the Family Adaptability and Cohesion Scales-III (FACES III; Olson et al., 1985), a 20-item instrument that can discriminate between distressed and non-distressed families; the Family Environment Scales (FES; Moos and Moos, 1981), a 90-item instrument assessing social climate; the Family Assessment Measure (FAM; Skinner et al., 1983), a 50-item scale differentiating between distressed and non-distressed families; and the Beavers Self-Report of Family Functioning (Beavers et al., 1988), a 34-item scale shown to discriminate between neglectful and non-neglectful families. In their discussion of five common measures of family assessment based on family systems theory (namely, Beavers System Model, FAD, MOOS, FAM, and FACES III), Franklin et al. (2004) assessed the reliability and validity of each measure and concluded that researchers must develop a comprehensive understanding of the psychometric development of different family measures to ensure the most appropriate one is used. The Family Assessment Device (FAD; Epstein et al., 1983) is another well-established measure of family functioning and differs from other measures in that it measures dimensions that are conducive to effective and healthy functioning of the family as a whole. These dimensions are (1) problem solving, (2) communication, (3) roles, (4) affective responsiveness, (5) affective involvement and (6) behavioral control.

Using the FAD, researchers have consistently distinguished between a ‘healthy family,’ which is described as having effective family functioning (e.g., the family’s ability to resolve problems) from an ‘unhealthy family,’ which is described as disturbed or having ineffective functioning (e.g., having difficulties achieving agreeable solutions that influence the family’s integrity; Miller et al., 2000a). The FAD has been found to be culturally acceptable (Shek, 2001; Speranza et al., 2012) and research supports its validity and reliability (Byles et al., 1988; Stevenson-Hinde and Akister, 1995; Bihun et al., 2002; Barney and Max, 2005; Georgiades et al., 2008). Importantly, it has been used in several studies that focused specifically on the link between child psychopathology and family functioning (Cunningham et al., 1988).

In addition to the six dimensions, a 12-item general functioning subscale (FAD-GF) was developed that assesses the “overall health and pathology of the family” (Epstein et al., 1983). The items of the FAD-GF subscale were developed to be multidimensional and correlate highly with the other FAD scales (Miller et al., 2000a). The FAD-GF subscale is increasingly used by researchers as the sole indicator of family functioning because it measures family functioning from the perspective of how well the family works together on essential tasks as an interrelated system (Speranza et al., 2012). The brevity of the scale also makes it suitable to use especially with children (Byles et al., 1988).

Only one study to date has addressed the question of whether the FAD can be used by children under 12 (Bihun et al., 2002). In this study, 132 children with asthma under the age of 12 (*M* = 9.7 years) were asked to fill in the FAD. A researcher was present in the room and used a series of clarifying statements for any child who required assistance. This study revealed that although reliabilities were lowest with younger children (Cronbach’s α = 0.48-0.79), there was good concurrent validity on measures of family functioning according to mothers’ reports. No study to date, however, has assessed the General Functioning subscale of the FAD when administered to school children younger than 12 years of age.

### Aims and Hypotheses

The main aim of the current study was to investigate whether family functioning affects children’s outcomes in internalizing symptomology. It is hypothesized (H1) that the FAD-GF subscale will be useful for gathering reliable and valid information about family functioning from school children under the age of 12 years.

Maternal/paternal perceptions and adolescent perceptions of family functioning have been implicated in internalizing symptomology; however, it remains unclear if and how a child’s perception of family functioning impacts on their internalizing symptoms. Given the importance and influence of family in a child’s life, it is hypothesized (H2) that a child’s perception of their family functioning at pre-test will predict their levels of internalizing symptoms at the 6-months follow-up. In addition, it remains unclear what influence perceptions of family function have on treatment outcomes for children with internalizing symptoms. Although it is expected that children who participate in the Aussie Optimism Positive Thinking Skills Program, a universal prevention program will report improvements in internalizing symptomology compared to the control group at the 6-months follow-up, it is hypothesized (H3) that those children who perceive their family as less healthy will report less improvement in internalizing symptomology.

## Materials and Methods

### Participants

Of the available (1118) year 4 and year 5 students, 847 (429 males, 418 females) from 13 primary school were given parental permission to be involved in the study and participated in the pre-test. There were five schools (281 students) in the control condition, and eight schools (607 students) in the intervention condition. The age range was 9–11 years. The range is reported because the students’ dates of birth were not collected.

### Research Design

Initially, selected schools were organized into pairs such that the members of each pair were matched in terms factors that might confound the intervention effects (e.g., school size); one member of each pair was then randomly assigned to the intervention condition while the other was assigned to the control condition. This strategy resulted in an equal number of schools in each condition. Unfortunately, several control schools dropped out prior to the pre-test resulting in an unequal distribution of schools across intervention and control conditions.

### Measures

The Family Assessment Device General Functioning Subscale (FAD-GF). The GF scale of the FAD, which measures overall health and pathology of the family, is recommended for use as a brief version of the FAD and is often used by researchers instead of the full FAD. The FAD-GF subscale is comprised of 12 statements that describe family behavior and relationships in each of the six dimensions. Six of the items are worded to describe healthy functioning (e.g., We are able to make decisions about how we solve problems), and six items are worded to describe unhealthy functioning (e.g., We cannot talk to each other about the sadness we feel). Each item belongs exclusively to the FAD-GF subscale but reflects the aspects of the other six FAD scales. The response option for each statement is (1) strongly agree, (2) agree, (3) disagree, and (4) strongly disagree. The four alternative responses are appropriate for the age group as it varies from best functioning (strongly agree) to worst functioning (strongly disagree) with no “neutral” option as this can be seen as an easy response option and may not represent the true answer (Robert, 1987). The FAD-GF subscale is scored by summing the endorsed responses (negatively worded items are reverse scored) and dividing by the number of items in the scale (Miller et al., 2000b). Miller et al. (1985) recommend a clinical cut-off score of 2 to discriminate ‘healthy’ from ‘unhealthy’ family functioning. The range for the scale scores varies from 1 (best functioning) to 4 (worse functioning; Miller et al., 2000b). They report a diagnostic confidence rating of 0.83 for this method. Epstein et al. (1983) reported that the FAD-GF subscale was internally consistent (Cronbach’s α = 0.92) and was able to discriminate between clinical and non-clinical samples (Shek, 2001). The FAD-GF subscale is highly correlated with each of the six dimensional scales (range *r* = 0.48–0.76). Although the device was designed for participants aged 12 and over, in this study the questionnaire was read aloud to the students by the trained research assistants and any difficult words were fully explained (e.g., “confide” means “to tell something to someone you trust”).

The Children Depression Inventory (CDI; Kovacs, 1992) was used to assess self-reported cognitive, affective and behavioral signs of depression in children aged 7–17. The CDI is a 27-item version of the Beck Depression Inventory, designed for use with children, and quantifies a range of depressive symptoms such as low mood, interpersonal difficulties, hedonic capacity and negative self-evaluations. Composite depression scores range from 0 to 54, with higher scores indicating more severe depressive symptomology. Two-week test–retest reliability and internal consistency coefficients have been reported to be above 0.80. Concurrent validity has been established with coefficients ranging from 0.66 to 0.81 (Kovacs, 1992). The suicide ideation item (Item 9) was removed from the scale as School Principals in the pilot study (Rooney et al., 2006) voiced concerns about the use of this item with children as young as 8 years. The total depression score in this study therefore ranged from 0 to 52, with higher scores indicating higher levels of depression symptoms. Rooney et al. (2006) reported a Cronbach’s alpha coefficient of 0.87 for the abbreviated CDI. Hodges (1990) used a clinical cut-off of 15 on the CDI for age groups similar to the one used in this study.

The Spence Children’s Anxiety Scale (SCAS; Spence, 1998) is designed to evaluate symptoms pertaining to various forms of anxiety such as separation anxiety, specific and social phobia, obsessive–compulsive disorder, panic-agoraphobia and generalized anxiety. Confirmatory factor analyses have supported the six proposed factors of the SCAS (Muris et al., 2002). In addition, the SCAS was found to discriminate well between anxious and community children, and between the different anxiety disorders. With respect to reliability, the following omega coefficients were obtained for the subscales: panic attack and agoraphobia: 0.82; separation anxiety disorder: 0.76; social phobia: 0.76; physical injury fears: 0.55; obsessive–compulsive disorder: 0.77; and generalized anxiety disorder: 0.80. Child reports were found to correlate well with parent reports for internalizing symptoms (*r* = 0.52). Analysis showed that the correlation between SCAS and the internalizing subscale (Withdrawn, Somatic complaints and Anxious/Depressed scales) of the Child Behavioral Checklist (CBCL; *r* = 0.27) was significantly higher than the correlation between the SCAS and CBCL’s externalizing subscales (Delinquent behavior and Aggressive Behavior domains; *r* = 0.12; *z* = 3.15, *p*< 0.001), providing evidence for both divergent and convergent validity.

### Other Measures

Variables used in the assessment of FASD-GF’s reliability and validity were chosen from the parent self-report questionnaire which included questions about the incidence of parents’ physical health problems in the last 12 months (a “yes” response required a further “please specify”), the incidence of mental health problems in the past 12 months (a “yes” response to this question required further response as to “what type of mental health problem?”); parental marital status (responses included single, de-facto, separated, divorced or widowed); physical health status of the parent; and family structure (single family, two parent family- blended and original, grandparent, other relative, legal guardian). Socioeconomic status (SES) was measured by the question “which statement best describes your family’s money situation?” with responses ranging from “we spend more than we get” to “we can save a lot” (1–5 point scale).

### Procedure

The study is part of the Enhanced Aussie Optimism Positive Thinking Program (Rooney et al., 2009). Thirteen private schools (eight intervention, five control) were selected from high socio-economic areas. All teachers attended a 1-day (8 h) training course, run by clinical psychologists who were members of the research team, in which they were instructed in running groups and the principles of “Positive Thinking.” Teachers in the intervention schools were provided with program manuals and resources. Following each session the teachers in the intervention schools assessed the program integrity and were offered a further 5 × 1 h coaching sessions. By June/July 2010, all participating schools received the assessment battery to provide a baseline pre-test measure of demographics, anxiety, depressive symptoms and family assessment. Participants who scored over 17 on the CDI or above 42 on the SACS were further followed up by post-graduate psychology students for assessment in a confidential manner to avoid stigmatization. This same procedure was repeated at post-test and at 6-months follow up. The 10 weekly intervention sessions were run in the usual classroom groups by the trained class teacher with support from the teacher assistant. The initial module focused on confidentiality followed by discussions on having fun, being brave, differentiating thoughts and feelings, thinking in a helpful way, coping skills and being positive. The average size of the classroom group was around 15 students per class.

Administration of the program was monitored by the chief investigators for adherence to the assigned intervention program via log books, reports, interviews, and observations. This was conducted by research assistants on all sessions to provide data to triangulate with the teacher log books. Apart from the integrity checklists, integrity of the program was maximized through observations by research assistants and friendly follow-up sessions to help teachers get through the program and by addressing any problems that arose. Dosage effect was measured via attendance of students at sessions.

The FAD-GF was used as a measure of family functioning. It was included because of the perceived importance and possible moderating influence of family functioning in the etiology, diagnosis and treatment of childhood internalizing symptoms. The questionnaire was read aloud to the children by trained Aussie Optimism research assistants.

### Data Analysis

The reliability and validity of the FAD-GF (H1) was tested using standard psychometric techniques: LISREL (version 8.8) was used to conduct a confirmatory factor analysis on the pre-test FAD-GF item scores; Cronbach’s alpha was computed to test internal consistency; the test-retest reliability between the pre-test FAD-GF scores and the 6-months follow-up FAD-GF scores was used a measure of temporal stability; convergent validity was tested with the control group data by correlating pre-test FAD-GF scores with 6-months follow-up anxiety and depression scores; and construct validity was assessed by examining the relationships between the FAD-GF subscale and each of the family variables specified above.

H2 was tested with a generalized linear mixed model (GLMM) as implemented through SPSS’S (Version 20) GENLINMIXED procedure. The GLMM represents a special class of regression model. The GLMM is ‘generalized’ in the sense that it can handle outcome variables with markedly non-normal distributions; the GLMM is ‘mixed’ in the sense that it includes both random and fixed effects (Bryk and Raudenbush, 1987). For the present GLMMs, there were two nominal random effects (student and school), one categorical fixed effect (group; intervention, control), one ordinal fixed effect (time: pre-test, 6-months follow-up), and the Group × Time interaction. In order to optimize the likelihood of convergence, a separate GLMM analysis was run for each of the outcomes (CDI, SCAS). The GLMM for testing H3 included the additional scale fixed effect of family functioning (FAD-GF) and the Group × FAD-GF, the Time × FAD-GF, and the Group × Time × FAD-GF interactions.

The traditional ANOVA model assumes normality, homogeneity of variance, and independence of observations. The GLMM ‘robust statistics’ option will generally take care of violations of normality and homogeneity of variance. Moreover, by specifying the multilevel nature of the current data (i.e., students nested within schools) in the GLMM, the model can accommodate intra-school dependencies in the outcome measures. GLMM is also robust to unequal group sizes. Finally, GLMM does not rely on participants providing data at every assessment point because the GLMM maximum likelihood procedure is a full information estimation procedure that uses all the data present at each assessment point. This reduces sampling bias and the need to replace missing data.

## Results

At pre-test, 580 intervention students and 269 control students were administered the SCAS and the CDI; and 444 intervention students and 207 control students were administered the FAD-GF. At the 6-months follow-up, 517 intervention students and 238 control students were administered the SCAS and the CDI; and 444 intervention students and 207 control students were administered the FAD-GF. The intervention group and the control group did not differ at pre-test in their reports of family functioning [*F*(1,649) = 1.47, *p* = 0.226] or of anxiety [*F*(1,847) = 2.75, *p* = 0.098], but there was a significant difference between the groups on depression scores [*F*(1,847) = 5.35, *p* = 0.021] with the control group reporting higher scores (*M* = 8.75) compared to the intervention group (*M* = 7.48). Overall the sample was a healthy one with total mean scores for the FAD-GF falling beneath the clinical cut off of 2 (*M* = 1.87, SD = 0.47). Mean and SD are presented in **Table 1**.

**Table 1:** Pre-test and 6-months follow up mean and SD for scores.

	Pre-test		6-months follow up	
	Control mean (SD)	Intervention mean (SD)	Control mean (SD)	Intervention mean (SD)
FAD-GD	1.90 (0.48)	1.85 (0.45)	1.89 (0.48)	1.85 (0.41)
CDI	8.64 (7.76)	7.52 (6.73)	6.69 (6.92)	5.08 (5.81)
SCAS	28.39 (15.86)	27.09 (15.42)	23.78 (15.31)	20.64 (13.04)

### Testing the Factorial Validity of the FAD-GF Using the Pre-Test Data

A range of fit indices was computed to determine how well the one-factor model fit the data (see **Table 2**). The comparative fit index (CFI) and the normed fit index (NFI) passed threshold (CFI = 0.92 > 0.90; NFI = 0.91 > 0.90), as did the standardized root mean square residual (SRMR = 0.06 < 0.08). The 90% CI for the root mean square error of approximation (RMSEA), however, failed to encompass 0.08 and therefore indicated a less than adequate fit. Tabachnick and Fidell (2001) note that the RMSEA has a tendency to over-reject the true model. Overall, therefore, the results indicate an acceptable fit for the one-factor solution.

**Table 2:** Fit statistics for the confirmatory factor analysis.

Comparative fit index (CFI)	Normed fit index (NFI)	Standardized root mean square residual (SRMR)	Root mean square error of approximation (RMSEA)
0.920	0.908	0.064	0.101 (90% CI: 0.092, 0.110)

***CFI:** a value greater than or equal to 0.90 indicates a good fit (Hu and Bentler, 1999)*.

***NFI:** a value greater than or equal to 0.90 indicates a good fit (Hu and Bentler, 1999)*.

***SRMR:** a value less than or equal to 0.08 indicates a good fit (Hu and Bentler, 1999)*.

***RMSEA:** a value less than or equal to 0.08, or a CI that encompass this value, indicates a good fit (Jaccard and Wan, 1996)*.

### Testing the Internal Consistency Reliability (Using the Pre-Test Data) and the Test–Retest Reliability (Using the Control Group Data) of the FAD-GF

Reliability of the FAD-GF scale was lower than previous findings. The internal consistency reliability (as measured by Cronbach’s alpha) was found to be 0.68, and the test–retest reliability was 0.47 (*p* < 0.001). For scales consisting of 5–9 items, 0.6 is acceptable; for scales with more than 10 items (like the FAD-GF) the desired value is 0.7 (Lowenthal, 2001).

### Testing the Convergent Validity of the FAD-GF Using the Pre-Test Data

Convergent validity is an aspect of construct validity. The FAD-GF has convergent validity if it is associated with other valid measures of family functioning such as health problems of parents, marital status, family structure, or family SES. The associations reported in **Table 3** provide no support for the convergent validity of the FAD-GF. The FAD-GF was, however, significantly correlated with both anxiety [*r*(*N* = 651) = 0.26, *p* < 0.001] and depression [*r*(*N* = 651) = 0.42, *p* < 0.001] at the pre-test assessments; although these correlations are too small to provide any convincing evidence for convergent validity.

**Table 3:** Associations (Eta-squared) between categorical criterion variables and FAD-GF scores.

Variable	Mean GF Scores		Eta-squared^*^	*p*
Mother psychological problem	Yes No	=1.85 =1.82	0.008	0.617
Mother depression	Yes No	=1.95 =1.75	0.002	0.695
Mother anxiety	Yes No	=1.85 =1.94	0.005	0.314
Father psychological problems	Yes No	=1.89 =1.86	0.001	0.962
Father aggressive	Yes No	=2.06^1^ =1.82	002	0.832
Mother marital status	Single Separated	=1.94 =1.85	0.007	0.809
Father marital status	Single Separated	=1.83 =1.85	0.004	0.916
Birth order	Sixth born	=2.65^1^	0.017	0.437
Family structure	Mum and dad Blended Mum only Dad only Grandparent Legal guardian	=1.87 =1.83 =1.80 =1.63 =2.04^1^ =1.67	0.005	0.936
Father physical health	Yes No	=1.79 =1.79	0.003	0.757
Mother physical health	Yes No	=1.70 =1.94	0.003	0.678
Family SES	Spend more than we get Just enough until payday Spend left over money We save a bit occasionally We can save a lot	=2.04^1^ =1.85 =1.80 =1.86 =1.83	0.013	0.414

^*^Conventions for defining eta-squared: 0.01 = small, 0.06 = moderate, 0.15+ = large.

*^1^Scores greater than two represent ‘unhealthy’ family functioning*.

### Testing the Predictive Validity of the FAD-GF Using the Control Group Data

Predictive validity is another aspect of construct validity. It was hypothesized that children’s perception of family functioning at pre-test would predict their level of anxiety and depression at the 6-months follow up. FAD-GF was a significant predictor of both depression (*b* = 5.10, *p* < 0.001, 95% CI = 2.80–7.40) and anxiety (*b* = 4.52, *p* < 0.001, 95% CI = 2.39–6.64) providing support for this aspect of construct validity.

### FAD-GF as a Moderator of the Intervention Effect

The intervention effect is embodied in the Group × Time interaction. This interaction was non-significant for both anxiety [*F*(1,1600) = 1.95, *p* = 0.163] and depression [*F*(1,1600) = 0.34, *p* = 0.560] indicating that the intervention had no impact on either anxiety or depression. There were, however, significant main effects for time indicating that both groups showed significant reductions in anxiety [*F*(1,1600) = 69.36, *p* < 0.001] and depression [*F*(1,1600) = 27.22, *p* < 0.001] between the pre-test and the 6-months follow-up.

The lack of intervention effects across the sample as a whole does not rule out the possibility of intervention effects for subgroups of individuals. H3, for instance, predicted that children’s perceptions of family functioning at pre-test would moderate the impact of the intervention on their levels of depression and anxiety, such that children who rated their family as less healthy at pre-test would benefit less from the intervention. The moderation effect is embodied in the FAD × Group × Time interaction. This interaction was non-significant for both anxiety [*F*(1,1198) = 0.08, *p* = 0.780] and depression [*F*(1,1198) = 0.41, *p* = 0.522] indicating that perceptions of family functioning at pre-test did not moderate the impact of the intervention on either anxiety or depression. There were, however, significant FAD × Time interactions for both anxiety [*F*(1,1198) = 16.90, *p* < 0.001] and depression [*F*(1,1198) = 20.21, *p* < 0.001] indicating that children with lower FAD scores at pre-test showed greater reduction in both anxiety and depression at 6 months regardless of the intervention.

## Discussion

One of the main purposes of this study was to determine whether the General Functioning Subscale of the FAD (Epstein et al., 1978), a brief self-report measure of overall family functioning, could be used with school children under the age of 12 in a universal prevention program targeting internalizing symptoms. The results from this study provide some evidence to indicate that the FAD-GF shows promise as a reliable and useful measure of a child’s perception of their family functioning in children as young as 9 years of age.

The results were mixed in terms of construct validity. One aspect of construct validity, convergent validity, was not established. Establishing convergent validity remains a difficult task because alternative measures of family functioning suitable for use by young children simply do not exist. Following Byles et al. (1988), this study used family variables considered to be associated with emotional and behavioral disorders in children in order to establish convergent validity. Unlike the Byles et al. (1988) study, however, the expected associations did not emerge. This may have been because the children were pre-adolescent and research has shown that the relationship between child internalizing and adult psychopathology becomes stronger as children get older (Connell and Goodman, 2002). Another aspect of construct validity, predictive validity, was established. This study found that children’s perception of family functioning at pre-test predicted their level of anxiety and depression at the 6-months follow-up indicating that the FAD-GF has good predictive validity for internalizing symptoms.

The internal consistency of the FAD-GF was 0.68, which was lower than expected. Lower internal consistencies have been reported previously with children under the age of 12. Bihun et al. (2002) used the full 60-item version of the FAD and reported unacceptable internal consistencies for some of the subscales (affective responsiveness, communication, behavior control and roles; Cronbach’s α = 0.48–0.60) but reported better internal consistency for the FAD-GF scale (Cronbach’s α > 0.65). Internal consistencies were notably lower for children under 12 years of age compared to children over 12 years of age. Stark et al. (1990) assessed perceived family functioning in children aged from 9 to 14 years (*M* = 11.69) with a self report measure of family functioning (SRMFF: Bloom, 1985) modified for children. Internal consistencies for the modified children’s measure (SRMFF-C) also tended to be in the unacceptable range (Cronbach’s α = 0.50–0.78).

There are several strategies for improving the internal consistency reliability of the FAD-GF for children under 12 years of age: (a) ensure that younger children are of an adequate reading level; (b) simplify the language and provide structured prompts or clarification for any difficulties experienced with the understanding of particular words in items (e.g, item 4: we *confide* in each other); (c) locate the FAD-GF questionnaire toward the beginning of a test battery in order to accommodate children who experience attention or fatigue difficulties and were not motivated to complete the task (in the present study, the FAD-GF was located toward the end of the questionnaires booklet).

Child report of family functioning as measured by the FAD-GF was a significant predictor of internalizing symptoms at 6 months. This finding is consistent with previous research. Stark et al. (1990) found that the diagnostic status of the child (mean age = 11.69) could be predicted by a knowledge of the child’s perceived family environment. Waller et al. (1990) found that adult daughter’s perceptions of family functioning as measured by the FAD was most predictive of the presence of family pathology and the presence or absence of an eating disorder. Kiliç et al. (2003) found that unhealthy family functioning as measured by the FAD-GF scale predicted state and trait anxiety in children aged 7–14 who had experienced an earthquake. These findings highlight the importance of understanding how children’s perceptions of their family life (perceived or actual) affect their internalizing symptoms.

This study did not find intervention effects for anxiety or depression. Participating in the AO-PTS Program had no significant effect on anxiety or depression at the 6-months follow-up. One possible explanation for the absence of intervention effects is that the participating students were all within the healthy range at pre-test; they were not identified as ‘at risk’ for depressive and anxiety symptoms, neither did they perceive their families as dysfunctional. This situation may have caused a ceiling effect reducing the possibility of gaining significant differences at the follow-up sessions. Another issue that may moderate intervention effects is dosage. The teacher log book, which contains the attendance list, activities and content covered, were not available to determine the amount of content delivered to the students. Trained teachers were either too busy to complete the log book or neglected to return the log book for assessment. Without the log book, it was not possible to determine whether the students received an appropriate dosage of the program and whether this reduced intervention effects at the 6-months follow-up.

Universal prevention programs targeting internalizing symptoms have consistently produced lower effect sizes compared to indicative and selective prevention programs (Teubert and Pinquart, 2011). This is not surprising given that many children develop well without receiving an intervention (Teubert and Pinquart, 2011). In this study, all children who were identified as ‘at risk’ (regardless of whether they were in the intervention or control condition) received written reports, possibly contaminating the intervention effect. The ‘at risk’ students were advised to consult their local GP or school psychologist for further treatment, which might explain why there were reduced numbers of depressive and anxiety symptoms at the 6-months follow-up for both intervention and control groups.

The lack of intervention effects across the sample as a whole does not rule out the possibility of intervention effects for subgroups of individuals. For instance, children’s perceptions of family functioning at pre-test might moderate the impact of the intervention on their levels of depression and anxiety, such that children who rated their family as less healthy at pre-test would benefit less from the intervention. There was no evidence that children’s perceptions of family functioning at pre-test moderated the intervention effect.

Of further interest was our finding that children with lower FAD-GF scores at pre-test showed a greater rate of reduction in both anxiety and depression at the 6-months follow-up. This suggests perceiving you’re in a healthy family may act as a protective factor against the development of internalizing symptoms.

### Limitations

Further research would benefit from investigating different reports of family functioning amongst sub groups of children such as gender, age groups (9, 10, and 11 year olds), high and low academic achievers and time in family structure to further our understanding of how family dysfunction affects what children think about their family life.

This current study targeted the high – socio economic areas where the independent and private schools are located as research has shown that private and small schools have the potential to contain aspects that may challenge students’ mental health (Watt, 2003). High social economic status families are described as physically and mentally healthier, financially, sociology and psychologically stable (Barnett, 2008; Australian Institute of Health and Welfare, 2012) but this study has found that children from high socio economic status families have shown some internalizing symptoms. Thus, it can be anticipated that more apparent outcomes will be obtained if a similar study was conducted within low socio economic areas with less social inclusion and ineffective family functioning, for example, stable housing and meaningful occupation, as these risk factors have been reported to be associated with mental illness (Commonwealth of Australia, 2009; Barnett and Hunter, 2012).

## Conclusion

Overall these finding suggests that the FAD-GF shows promise as a useful measure of family functioning in children as young as 9 years of age. This has important implications for future research in which ease of administration and cost are also key factors. Further, these results indicate that a child’s rating of the overall health of their family may play an important role in identifying children at risk for internalizing disorders. That is, it may be that the child’s perception of their family functioning and not the actual family functioning that is important in predicting internalizing symptoms.

## Conflict of Interest Statement

The authors declare that the research was conducted in the absence of any commercial or financial relationships that could be construed as a potential conflict of interest.
